# Strategies to reduce sample sizes in Alzheimer’s disease primary and secondary prevention trials using longitudinal amyloid PET imaging

**DOI:** 10.1186/s13195-021-00819-2

**Published:** 2021-04-19

**Authors:** Isadora Lopes Alves, Fiona Heeman, Lyduine E. Collij, Gemma Salvadó, Nelleke Tolboom, Natàlia Vilor-Tejedor, Pawel Markiewicz, Maqsood Yaqub, David Cash, Elizabeth C. Mormino, Philip S. Insel, Ronald Boellaard, Bart N. M. van Berckel, Adriaan A. Lammertsma, Frederik Barkhof, Juan Domingo Gispert

**Affiliations:** 1 Department of Radiology and Nuclear Medicine, Amsterdam UMC, Vrije Universiteit Amsterdam, Amsterdam, The Netherlands; 2 Barcelonaβeta Brain Research Center, Pasqual Maragall Foundation, Barcelona, Spain; 3 IMIM (Hospital del Mar Medical Research Institute), Barcelona, Spain; 4 Imaging Division, Department of Radiology, University Medical Center Utrecht, Utrecht, The Netherlands; 5 Centre for Genomic Regulation (CRG), The Barcelona Institute for Science and Technology, Barcelona, Spain; 6 Department of Clinical Genetics, Erasmus MC University Medical Center Rotterdam, Rotterdam, The Netherlands; 7 Universitat Pompeu Fabra, Barcelona, Spain; 8 Centre for Medical Image Computing, Medical Physics and Biomedical Engineering, UCL, London, UK; 9 Dementia Research Centre, UCL Queen Square Institute of Neurology, London, UK; 10 Department of Neurology and Neurological Sciences, Stanford University, Stanford, CA USA; 11 Clinical Memory Research Unit, Faculty of Medicine, Lund University, Lund, Sweden; 12 Department of Psychiatry, University of California, San Francisco, CA USA; 13 Centro de Investigación Biomédica en Red Bioingeniería, Biomateriales y Nanomedicina, Madrid, Spain

**Keywords:** PET imaging, Amyloid, Alzheimer’s disease, Prevention, Sample size, Clinical trial

## Abstract

**Background:**

Detecting subtle-to-moderate biomarker changes such as those in amyloid PET imaging becomes increasingly relevant in the context of primary and secondary prevention of Alzheimer’s disease (AD). This work aimed to determine if and when distribution volume ratio (DVR; derived from dynamic imaging) and regional quantitative values could improve statistical power in AD prevention trials.

**Methods:**

Baseline and annualized % change in [^11^C]PIB SUVR and DVR were computed for a global (cortical) and regional (early) composite from scans of 237 cognitively unimpaired subjects from the OASIS-3 database (www.oasis-brains.org). Bland-Altman and correlation analyses were used to assess the relationship between SUVR and DVR. General linear models and linear mixed effects models were used to determine effects of age, sex, and *APOE*-ε4 carriership on baseline and longitudinal amyloid burden. Finally, differences in statistical power of SUVR and DVR (cortical or early composite) were assessed considering three anti-amyloid trial scenarios: secondary prevention trials including subjects with (1) intermediate-to-high (Centiloid > 20.1), or (2) intermediate (20.1 < Centiloid ≤ 49.4) amyloid burden, and (3) a primary prevention trial focusing on subjects with low amyloid burden (Centiloid ≤ 20.1). Trial scenarios were set to detect 20% reduction in accumulation rates across the whole population and in *APOE*-ε4 carriers only.

**Results:**

Although highly correlated to DVR (*ρ* = .96), cortical SUVR overestimated DVR cross-sectionally and in annual % change. In secondary prevention trials, DVR required 143 subjects per arm, compared with 176 for SUVR. Both restricting inclusion to individuals with intermediate amyloid burden levels or to *APOE*-ε4 carriers alone further reduced sample sizes. For primary prevention, SUVR required less subjects per arm (*n* = 855) compared with DVR (*n* = 1508) and the early composite also provided considerable sample size reductions (*n* = 855 to *n* = 509 for SUVR, *n* = 1508 to *n* = 734 for DVR).

**Conclusion:**

Sample sizes in AD secondary prevention trials can be reduced by the acquisition of dynamic PET scans and/or by restricting inclusion to subjects with intermediate amyloid burden or to *APOE*-ε4 carriers only. Using a targeted early composite only leads to reductions of sample size requirements in primary prevention trials. These findings support strategies to enable smaller Proof-of-Concept Phase II clinical trials to better streamline drug development.

**Supplementary Information:**

The online version contains supplementary material available at 10.1186/s13195-021-00819-2.

## Background

With the recently established biological definition of Alzheimer’s disease (AD) [1] and the increased availability of (imaging) biomarkers, the research community is now well-equipped to study this disease from its earliest pathological changes to later-stage clinical presentations of cognitive impairment [2]. Especially in the context of much needed treatment and prevention strategies, this research framework can be extremely valuable in accurately identifying individuals in the AD *continuum*, who might benefit from disease-modifying therapies.

With varying degrees of pathological confirmation, recent years have seen many disease-modifying therapies that failed to meet primary endpoints and impact cognitive functioning [3]. In fact, despite promising signals observed in a number of anti-amyloid clinical trials [4–8], the lack of downstream effects on cognition posed important questions on the validity of the widely accepted amyloid cascade hypothesis and highlighted our (still) limited understanding of the mechanisms involved in this disease. Nonetheless, recent results such as those from the aducanumab [7, 9, 10] or BAN2410 [11] trials have shown promising signals for anti-amyloid therapies and in fact have encouraged the development of earlier preventive Phase 3 trials focusing on subjects with preclinical AD such as the AHEAD 3-45 Study [12]. As a result, this shift to prevention in earlier stages of the disease and the (possible) future need for pathological confirmation pre-treatment may increase the use of biomarkers such as amyloid positron emission tomography (PET) imaging for both screening and measurement of treatment effects. However, a marked discrepancy in duration between most short-term studies and the long-term pathological processes such as Aβ plaque accumulation [13, 14] may result in the need to detect subtle-to-moderate biomarker changes [15].

When focusing on the early stages of AD with amyloid PET, observed changes in Aβ burden are mostly focal [16–18], and it may be difficult to detect these changes with sufficient statistical power, challenging standard analytical approaches, and the traditional use of a global measure of amyloid burden [19, 20]. Recent work suggests that regional amyloid PET assessments can improve early detection of pathology [16, 21, 22] and achieve increased power in clinical trials [23]. In addition, several PET studies have investigated potential methodological improvements to increase statistical power in longitudinal settings and better discriminate sub-populations cross-sectionally [24]. These studies generally focused on improving technical factors affecting image quality such as partial volume effects [25] or on modeling and pre-processing choices impacting measurement stability, such as the choice of reference region [26–28]. However, since the vast majority of PET studies performs static acquisitions, these improvements remain mostly limited to the use of the standard uptake value ratio (SUVR) metric. Although easily available from short static scans, SUVR is a semi-quantitative and biased proxy of the specific amyloid burden as measured by binding potential (*BP*_ND_) or distribution volume ratio (DVR) [29, 30], which are available only from dynamic scans. Specifically, SUVR is known to suffer from technical and physiological sources of bias such as inconsistent scanning window and changes in cerebral blood flow [30, 31]. However, traditional dynamic acquisitions can significantly increase the duration and cost of studies; therefore, compromises have been proposed, such as the collection of early frames in addition to the standard late-uptake image acquisition [32]. In fact, this early frame collection not only allows for the determination of DVR, but provides an additional parameter (*R*_1_) that can serve as a proxy for cerebral blood flow, another important marker of disease in AD [33, 34].

Considering current and future research needs, this study aims to determine if and when dynamic imaging and targeted regional quantification could improve statistical power in primary and secondary prevention trials using longitudinal amyloid PET imaging. For that purpose, we estimated the number of participants per arm needed in three hypothetical trial scenarios aiming to reduce amyloid accumulation rates by at least 20%: (1) one in subjects with low amyloid burden for primary prevention, and two for secondary prevention, either (2) including all subjects with abnormal amyloid levels (intermediate-to-high) or (3) focusing on those at the earliest stages of pathology (intermediate levels). We compared the sample sizes required when using SUVR and DVR as amyloid load metric in both the whole population as well in trial scenarios only recruiting *APOE*-ε4 carriers.

## Methods

### Data sets

This work included two separate datasets: the first was used for main analyses, and the second for calculating test-retest variability for [^11^C]PIB SUVR and DVR.

For the first dataset, tabulated PET data were obtained from the Open Access Series of Imaging Studies (OASIS-3) dataset, which is a longitudinal neuroimaging, clinical, cognitive, and biomarker dataset for normal aging and Alzheimer’s disease (www.oasis-brains.org). This dataset is a retrospective compilation of data collected across several ongoing projects through the Washington University of Saint Louis Knight Alzheimer’s Disease Research Center (ADRC) over the course of 30 years [35]. A total of 237 subjects were selected based on (1) being classified as cognitively unimpaired and (2) having at least two dynamic [^11^C]PIB PET scans with a minimum of 1 year between sessions available.

For the second dataset, eleven subjects (4 cognitively unimpaired, 1 mild cognitive impaired, and 6 with AD dementia) were selected from a previously reported test-retest (TRT) study at the Amsterdam University Medical Center location VUmc [36]. Test and retest scans were performed within a one week interval.

### Image acquisition and processing

A brief description of data collection and standard imaging processing pipelines for each dataset can be found below.

OASIS-3 60 min dynamic [^11^C]PIB PET images were acquired starting at the intravenous administration of approximately 12 mCi of radiotracer. Data was collected in 3D mode on a Siemens/CTI EXACT HR+ scanner or a Biograph 40 PET/CT scanner. Accompanying anatomical T1-weighted MPRAGE MR scans were acquired using either a Siemens 1.5 of 3T scanner. Image processing was performed with a local processing pipeline (PUP; https://github.com/ysu001/PUP), described in detail previously [37]. In short, the standard FreeSurfer (v5.3; Martinos Center for Biomedical Imaging, Charlestown, Massachusetts, USA; https://surfer.nmr.mgh.harvard.edu/fswiki) based PUP processing includes a scanner resolution harmonization filter [38], inter-frame motion correction, PET-MR registration, and regional time-activity curves extraction for all regions from the Desikan-Killiany atlas (DK) [39]. Using the cerebellar cortex as the reference region, reference Logan graphical analysis (RLogan) [40] was used to determine DVR with t* set to 30 min post-injection (p.i.). In parallel, SUVR was extracted for the same time-window of 30–60 min p.i.

For the TRT study, 90 min dynamic [^11^C]PIB PET scans were performed on a Siemens ECAT EXACT HR+ scanner and a structural T1-weighted MR scan on a 1.5 T Siemens Sonata scanner. First, structural T1-weighted MR images were co-registered to the PET scan using Vinci software (Max Planck Institute for Neurological Research, Cologne, Germany) and PVE-lab software was used to extract the cerebellar cortex time-activity curve based on the Hammers atlas [41, 42]. Next, both DVR (RLogan) and SUVR were calculated from 30 to 60 min p.i. in order to compare results with those from the OASIS-3 dataset and finally normalized to the cerebellar cortex using PPET software [43]. These parametric images were then warped into MNI space using SPM12 and the DK atlas was used to extract regional SUVR and DVR values.

Both global and regional analyses were performed on the SUVR and DVR data. A global measure of amyloid burden was determined based on a “cortical composite” created from grey-matter FreeSurfer-defined frontal, parietal, temporal, and precuneus regions [37]. In addition, an “early composite” was defined from three grey-matter DK regions, namely the isthmus cingulate, precuneus, and lateral orbitofrontal cortices. These regions were chosen based on literature for consistently displaying increased amyloid burden in early disease stages, as well as higher rates of accumulation compared with cortical composites [16–19, 44]. Finally, corresponding and previously validated Centiloid (CL) values were also available for comparison in the OASIS-3 dataset [26].

### Levels of β-amyloid burden

Three different levels of amyloid burden were defined based on CL cutoffs available from literature and validated against pathology [45]. Low amyloid burden was defined as CL values below 20.1, a threshold showing the highest accuracy in detecting moderate or frequent plaque density. In contrast, high amyloid burden was defined as CL values above 49.4, the threshold found to identify intermediate or high likelihood of Alzheimer’s disease according to NIA-AA 2012 criteria [46]. Finally, intermediate levels were those with 20.1 < CL ≤ 49.4.

### Amyloid accumulation

In order to account for differences in number of scans and interval between visits, a linear mixed effects model (LME) with random intercepts and random slopes was used to determine annualized rates of Aβ accumulation for every metric (SUVR and DVR) in the OASIS-3 dataset. To facilitate interpretability when reporting results, these were also normalized to baseline Aβ levels and will be reported as annualized % change.

Next, the TRT variability of each quantitative metric derived from the TRT dataset was used as a cutoff to determine the proportion of subjects to be considered as “accumulators,” i.e., those with annualized % change above TRT variability. Relative TRT variability was calculated for all subjects from the TRT dataset (*n* = 11) and for cognitively unimpaired subjects only (*n* = 4), according to Eq. 1, where the estimate of amyloid burden (DVR or SUVR) of the test scan is denoted as *T* and for the retest scan as *R*.

1 $$ \mathrm{T}\mathrm{RT}\ \left(\%\right)=\frac{\mid \mathrm{T}-\mathrm{R}\mid }{0.5\bullet \mid \mathrm{T}+\mathrm{R}\mid}\bullet 100 $$

### Statistical analysis

All statistical analysis were performed using R Statistical Software (version 4.0.2; R Foundation for Statistical Computing, Vienna, Austria). Results are reported as mean ± standard deviation (μ ± SD) or median (M) and interquartile range (IQR), as appropriate. In all analyses, DVR was considered the reference metric.

To assess the relationship between cortical SUVR and DVR at baseline and longitudinally, Bland-Altman plots, correlation analyses, and paired *t* tests (or Wilcoxon signed-rank test) were used. In addition, paired *t* tests (or Wilcoxon signed-rank test) were also used to assess differences between a cortical composite and an early composite in the estimation of amyloid burden and accumulation rates.

To assess the relationship between baseline amyloid burden and longitudinal amyloid accumulation, a linear, a quadratic and a natural cubic spline model with 1 knot were tested, and the optimal model was determined based on the Akaike information criteria (AIC).

Finally, effects of age, *APOE*-ε4 carriership (presence of at least 1 ε4 allele), and sex on baseline amyloid burden were assessed by a general linear model (GLM). Similarly, a linear mixed effects model (LME) was used to determine the effect of the same variables on amyloid accumulation, accounting for baseline amyloid burden.

The analyses above were performed in order to determine the generalizability of the OASIS-3 dataset with respect to other cohorts, such that the results of the sample size calculations can be contextualized appropriately.

### Sample size calculations

Using the LME estimates for annualized accumulation rates and respective standard deviations, the *sampsizepwr* function in Matlab (1-β = 80% power and a two-tailed *t* test type-I error of *α* = 0.05) was used to determine sample sizes required to detect differences in accumulation rates in three hypothetical 12-month placebo-controlled randomized anti-amyloid clinical trials. The trial designs assumed participants undergo a PET scan at baseline and another at the completion of the trial. These were computed separately for SUVR and DVR, using the cortical composite and the early composite, both across the whole population and restricted to *APOE*-ε4 carriers only.

The tested trial scenarios were the following:

1. A secondary prevention trial aiming to detect a 20% reduction in β-amyloid accumulation rates in individuals with intermediate-to-high amyloid burden (CL > 20.1) at baseline;
2. An *earlier* secondary prevention trial aiming to detect a 20% reduction in β-amyloid accumulation rates focusing in individuals with intermediate amyloid burden (20.1 < CL ≤ 49.4) at baseline;
3. A primary prevention trial aiming to detect a 20% reduction in in β-amyloid accumulation rates in individuals with low amyloid burden (CL ≤ 20.1) at baseline.

## Results

On average, OASIS-3 subjects underwent 2.5 ± 0.6 scans [range 2–5], with an average of 4.8 ± 2.1 years between the first and the last scan [range 1–9.6]. The majority of subjects were female (65.0%), 32.9% of them were *APOE*-ε4 carriers, and the mean age at the time of the first PET session was 65.3 ± 9.4 years. Complete OASIS-3 cohort demographics are shown in Table 1.

**Table 1:** Descriptive composition of included subjects in terms of baseline amyloid status, *APOE*-ε4 carriership, sex, age, and annualized accumulation rates in DVR and SUVR

	Whole cohort			Low burden group (CL ≤ 20.1)			Intermediate burden group (20.1 < CL ≤ 49.4)			High burden group (CL > 49.4)		
	All	*APOE*-ε4 non-carriers	*APOE*-ε4 carriers	All	*APOE*-ε4 non-carriers	*APOE*-ε4 carriers	All	*APOE*-ε4 non-carriers	*APOE*-ε4 carriers	All	*APOE*-ε4 non-carriers	*APOE*-ε4 carriers
Number of subjects (%)	237 (100)	159 (67.1)	78 (32.9)	194 (81.8)	146 (75.2)	48 (24.8)	20 (8.44)	5 (25.0)	15 (75.0)	23 (9.70)	8 (34.8)	15 (65.2)
Number of women (%)	154 (65.0)	100 (62.9)	54 (69.2)	129 (66.5)	95 (65.1)	34 (70.8)	13 (65.0)	1 (20.0)	12 (80.0)	12 (52.2)	4 (50.0)	8 (53.3)
Age, years	65.3 ± 9.4	65.8 ± 9.5	64.5 ± 9.2	64.4 ± 9.6	65.3 ± 9.7	61.7 ± 9.2	67.6 ± 7.4	67.7 ± 3.9	67.6 ± 8.3	71.1 ± 6.6	72.4 ± 7.0	70.3 ± 6.5
**SUVR (cortical composite)**												
Baseline (IQR)	1.08 (1.05–1.14)	1.07 (1.05–1.12)	1.15 (1.05–1.44)	1.07 (1.04–1.11)	1.07 (1.04–1.10)	1.07 (1.04–1.14)	1.40 (1.29–1.45)	1.38 (1.30–1.47)	1.41 (1.28–1.45)	1.73 (1.61–1.91)	1.79 (1.59–1.91)	1.72 (1.61–1.91)
Annual % change	1.06 (1.30)	0.77 (1.08)	1.63 (1.53)	0.80 (1.18)	0.69 (0.99)	1.15 (1.56)	2.83 (0.93)	2.81 (0.69)	2.84 (1.02)	1.77 (1.18)	2.37 (1.34)	1.98 (1.07)
Effect size	0.76	0.69	1.10	0.69	0.63	0.72	3.54	4.75	3.00	1.58	1.05	1.89
Number of accumulators (%) [CUs TRT]	45 (23.6)	25 (15.7)	31 (39.7)	27 (13.9)	17 (11.6)	10 (20.8)	17 (85.0)	5 (100.0)	12 (80.0)	12 (52.2)	3 (37.5)	9 (60.0)
Number of accumulators (%) [ALL TRT]	20 (8.4)	5 (3.1)	15 (19.2)	10 (5.2)	3 (2.1)	7 (14.6)	7 (35.0)	1 (20.0)	6 (40.0)	3 (13.0)	1 (12.5)	2 (13.3)
**SUVR (early composite)**												
Baseline (IQR)	1.16 (1.08–1.23)	1.14 (1.11–1.18)	1.22 (1.13–1.54)	1.14 (1.11–1.18)	1.14 (1.11–1.17)	1.15 (1.11–1.20)	1.50 (1.41–1.54)	1.50 (1.42–1.53)	1.50 (1.38–1.55)	1.79 (1.73–1.99)	1.89 (1.78–2.03)	1.79 (1.71–1.98)
Annual % change	1.25 (1.26)	0.99 (1.08)	1.75 (1.44)	1.02 (1.16)	0.91 (1.03)	1.34 (1.44)	2.87 (1.02)	2.74 (0.90)	2.90 (1.08)	1.74 (1.13)	1.36 (1.17)	1.94 (1.09)
Effect size	0.94	0.86	1.20	0.86	0.87	0.94	3.23	3.41	3.00	1.55	1.18	1.84
**DVR (cortical composite)**												
Baseline (IQR)	1.05 (1.02–1.10)	1.04 (1.01–1.07)	1.10 (1.03–1.30)	1.04 (1.01–1.06)	1.04 (1.01–1.06)	1.04 (1.02–1.09)	1.26 (1.21–1.30)	1.29 (1.21–1.34)	1.25 (1.20–1.30)	1.54 (1.43–1.69)	1.59 (1.44–1.71)	1.53 (1.43–1.69)
Annual % change	0.75 (1.11)	0.50 (0.91)	1.25 (1.30)	0.49 (0.96)	0.40 (0.82)	0.75 (1.29)	2.34 (0.72)	2.53 (0.43)	2.34 (0.80)	1.55 (0.93)	1.16 (1.07)	1.76 (0.80)
Effect size	0.69	0.54	1.00	0.48	0.50	0.57	3.75	7.11	3.22	1.86	1.06	2.25
Number of accumulators (%) [CUs TRT]	81 (34.2)	39 (24.5)	42 (53.8)	44 (22.7)	29 (19.9)	15 (31.3)	20 (100.0)	5 (100.0)	15 (100.0)	17 (73.9)	5 (62.5)	12 (80.0)
Number of accumulators (%) [ALL TRT]	33 (13.9)	11 (6.9)	22 (28.2)	13 (6.7)	6 (4.1)	7 (14.6)	13 (65.0)	4 (80.0)	9 (60.0)	7 (30.4)	1 (12.5)	6 (40.0)
**DVR (early composite)**												
Baseline (IQR)	1.11 (1.08–1.17)	1.10 (1.07–1.13)	1.16 (1.09–1.38)	1.09 (1.07–1.12)	1.09 (1.07–1.12)	1.10 (1.07–1.14)	1.34 (1.31–1.39)	1.39 (1.32–1.41)	1.34 (1.27–1.38)	1.66 (1.54–1.77)	1.72 (1.56–1.82)	1.59 (1.51–1.70)
Annual % change	0.94 (1.08)	0.72 (0.94)	1.34 (1.22)	0.71 (0.97)	0.63 (0.87)	0.94 (1.19)	2.49 (0.76)	2.55 (0.71)	2.47 (0.80)	1.50 (0.89)	1.15 (0.96)	1.68 (0.83)
Effect size	0.78	0.72	1.12	0.72	0.70	0.76	3.67	3.89	3.43	1.71	1.19	2.17

Values are described as mean ± standard deviation unless otherwise indicated. CU stands for cognitively unimpaired

Similarly, the cognitively unimpaired individuals from the TRT dataset (*n* = 4) were mainly female (75.0%), 33.3% were *APOE*-ε4 carriers, and their mean age was 66.8 ± 4.1 years. In contrast, the full dataset (*n* = 11) had a higher proportion of *APOE*-ε4 carriers (62.5%), equivalent proportion of males and females (45.5% females), and a mean age of 64.0 ± 4.9 years. Of note, *APOE* genotyping was missing for 3/11 subjects.

### Cortical and regional β-amyloid quantification

**Fig. 1:**
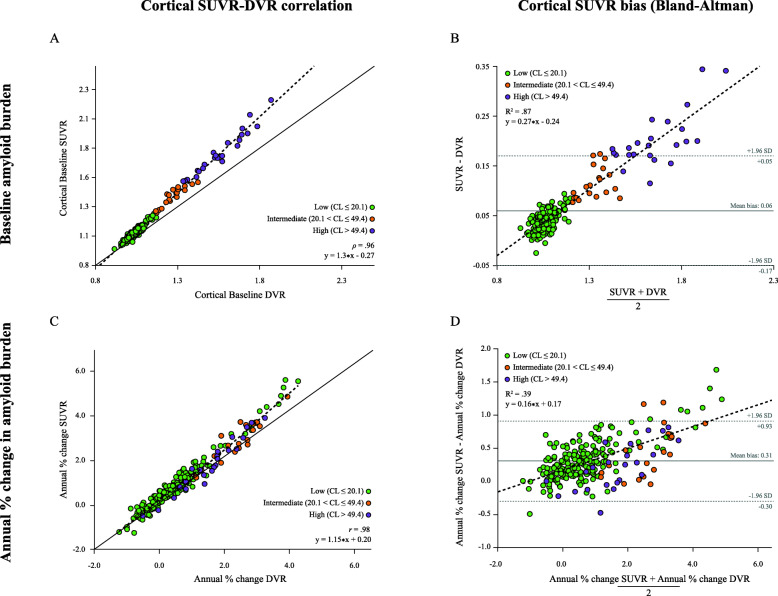
Relationship between SUVR and DVR. On the top panel, a scatterplot between baseline cortical SUVR and DVR across all subjects, with a solid identity line as reference (**a**), and a Bland-Altman plot displaying a linear relationship between SUVR bias and underlying amyloid burden (**b**). On the bottom panel, a scatterplot between annualized % cortical SUVR and DVR across all subjects, with a solid identity line as reference (**c**), and a Bland-Altman plot displaying a linear relationship between bias in annualized % cortical SUVR and underlying accumulation rates, with a dotted line representing a linear regression through the data points (**d**)

Baseline cortical SUVR (M = 1.08, IQR = 1.14–1.05) and DVR (M = 1.05, IQR = 1.10–1.02) were highly correlated (*ρ* = .96), and SUVR consistently overestimated DVR (Fig. 1a). Assuming DVR as the reference standard, this bias in SUVR was proportional to the underlying level of amyloid burden, where an increase in one amyloid burden unit translated to a 30% increase in bias (slope in Fig. 1b). The same pattern was found for annual % change, where the two metrics were highly correlated (*r* = .98, Fig. 1c), but SUVR (*μ* = 1.06 ± 1.30%) overestimated DVR-based accumulation rates (*μ* = 0.75 ± 1.11%) by 15% at every unit increase in underlying accumulation rates (slope in Fig. 1d).

**Fig. 2:**
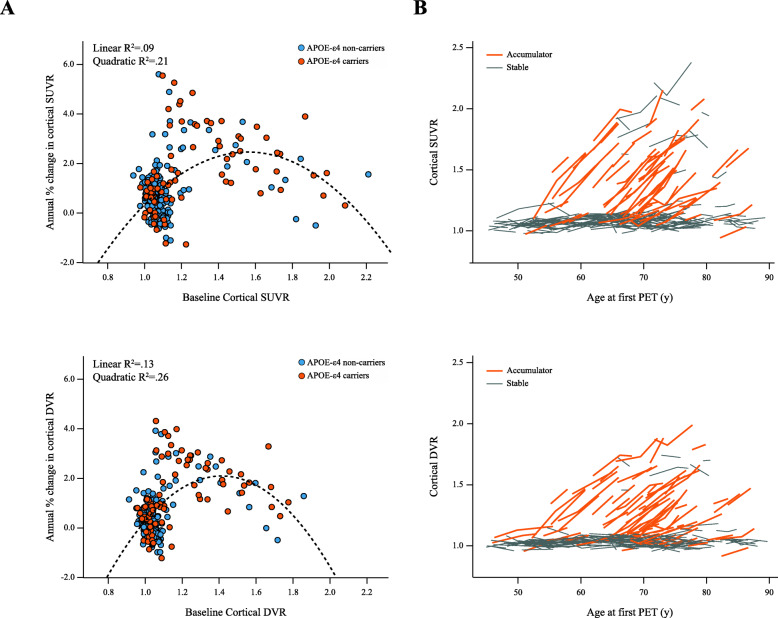
Amyloid accumulation with SUVR and DVR. Scatter plot of the relationship between annual % change and baseline amyloid levels using SUVR (top left) and DVR (bottom left) in *APOE*-ε4 carriers (orange) and non-carriers (blue), with a dotted line representing the quadratic model fit (**a**). Plot of the absolute change in SUVR (top right) and DVR (bottom right) in time, coded for whether subjects were classified as accumulators based on TRT from cognitively unimpaired individuals (orange) or were considered stable (gray) (**b**)

The relationship between baseline and longitudinal cortical amyloid burden was well described by a quadratic model for both SUVR (*R*^2^ = .21, ∆AIC_linear_ = − 94.4, ∆AIC_spline_ = − 95.3) and DVR (*R*^2^ = .26, ∆AIC_linear_ = − 78.8, ∆AIC_spline_ = − 79.2) (Fig. 2a), where subjects in the intermediate amyloid burden group displayed the highest accumulation rates on average (Table 1). Across the whole cohort, baseline   SUVR and DVR and respective annual % change did not differ between males and females, while higher age was associated with higher baseline SUVR (*β* = 0.007, *t* = 4.60, *p* < .001) and DVR (*β* = 0.005, *t* = 4.17, *p* < .001), but did not predict accumulation rates. Similarly, *APOE*-ε4 carriership was associated with higher baseline levels of amyloid burden (SUVR: *β* = 0.163, *t* = 5.69, *p* < 0.001; DVR: *β* = 0.122, *t* = 5.53, *p <* 0.001), and was only related to higher accumulation rates when using SUVR (*β* = 0.014, *t* = 2.55, *p* = .011).

As expected, both baseline and accumulation rates with SUVR and DVR were significantly higher when using the early composite compared to the cortical composite (Table 1).

### TRT and longitudinal amyloid accumulation

In order to assess the proportion of OASIS-3 participants with accumulation rates beyond TRT variability, we determined cutoffs for accumulation based on a separate local TRT dataset.

Focusing on cognitively unimpaired individuals only (*n* = 4), SUVR TRT was 1.61% for the cortical composite, compared to 0.85% for DVR. Similarly, a TRT of 3.46% was observed for SUVR with an early composite, while for the same ROI, DVR TRT was only 2.05%. In addition, when assessing subjects across diagnostic groups (*n* = 11), the pattern remains, with DVR TRT always lower than SUVR (cortical composite: 2.12% DVR/3.45% SUVR, early composite: 2.14% DVR/4.16% SUVR).

Using TRT from cognitively unimpaired subjects as our main cutoff for accumulation (due to cohort comparability) and a cortical composite for quantification, 81 (34.2%) individuals were classified as accumulators using DVR compared with 45 (23.6%) using SUVR (Fig. 2b). A total of 25 subjects were accumulators with DVR but not SUVR; 17 of them belonging to the low, 3 to the intermediate, and 5 to the high amyloid burden group (Table 1). Similarly, using the early composite for quantification and TRT cutoff, SUVR analyses classified 8 (3.4%) of subjects as accumulators compared to 39 (16.5%) when using DVR. In this case, 31 subjects were accumulators with DVR but not with SUVR, 10 of which were from the low, 15 from intermediate, and 6 from the high amyloid burden group.

### Sample sizes in longitudinal studies

Table 2 summarizes the required sample sizes for three hypothetical trial scenarios, considering different choices with respect to acquisition protocol (static/SUVR or dynamic/DVR), methodological (cortical composite or early composite), and inclusion criteria (whole population or *APOE*-ε4 carriers only).

**Table 2:** Sample size requirements per trial arm, for three hypothetical trial scenarios, comparing differences between using DVR/SUVR, a cortical/early composite ROI, and restricting the inclusion to *APOE*-ε4 carriers or not

	*Whole population*				*APOE- ε4 carriers only*			
	*SUVR*		*DVR*		*SUVR*		*DVR*	
	*Cortical ROI*	*Early ROI*	*Cortical ROI*	*Early ROI*	*Cortical ROI*	*Early ROI*	*Cortical ROI*	*Early ROI*
*Secondary prevention to detect 20% reduction in accumulation (CL > 20.1)*	*176*	*167*	*143*	*140*	*116*	*125*	*83*	*97*
*Early secondary prevention to detect 20% reduction in accumulation (20.1 < CL ≤ 49.4)*	*44*	*51*	*39*	*38*	*52*	*56*	*47*	*43*
*Primary prevention to detect 20% reduction in accumulation (CL ≤ 20.1)*	*855*	*509*	*1508*	*734*	*724*	*455*	*1162*	*630*

For secondary prevention trials aiming to detect a 20% reduction in β-amyloid accumulation rates, the sample sizes required are consistently lower when using DVR compared to SUVR (Table 2), likely because the smaller standard deviation and better TRT observed with DVR outweighs its lower average rate of accumulation (Table 1). In addition, including only *APOE*-ε4 carriers provided considerable reduction in the required sample sizes (whole population: *N*_SUVR_ = 176, *N*_DVR_ = 143, *APOE*-ε4 carriers only: *N*_SUVR_ = 116, *N*_DVR_ = 83), for either region of interest chosen for analysis. Further, if this secondary prevention trial included only individuals at an earlier stage of the disease (i.e., those with intermediate amyloid burden and thus more likely to have higher accumulation rates), a 4-fold reduction in required sample sizes (*N*_SUVR_ = 44, *N*_DVR_ = 39) can be achieved compared to including subjects from the general population (*N*_SUVR_ = 176, *N*_DVR_ = 143). In both secondary prevention scenarios, the use of an early composite did not reduce the required sample sizes.

**Fig. 3:**
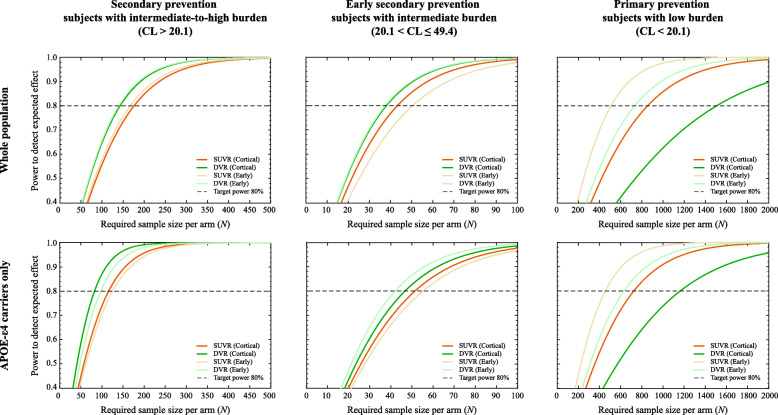
Sample size requirements (per arm). Relationship between achieved statistical power and number of participants required in three anti-amyloid hypothetical trial scenarios for the general population (top row) or focusing on *APOE*-ε4 carriers only (bottom row). The dotted line represents the desired power of 1-β = 80%

Finally, a primary prevention trial required the largest sample sizes overall as expected, and the use of an early composite reduced the number of subjects needed to detect the desired effect by ~ 40–50%, in case of both SUVR (*N*_CORTICAL_ = 855, *N*_EARLY_ = 509) and DVR (*N*_CORTICAL_ = 1508, *N*_EARLY_ = 734). Similarly, restricting the trial to *APOE*-ε4 carriers provided approximately ~ 20% reductions in sample size requirements with either acquisition protocol. However, in this scenario, the use of SUVR provided smaller sample size requirements than DVR (Fig. 3), which relates to its higher accumulation rates and similar standard deviation (Table 1).

## Discussion

In this work, we observed that the smaller variability of DVR compared with SUVR results in smaller sample size requirements for anti-amyloid secondary prevention trials when using dynamic amyloid PET scans. In addition, focusing on individuals with intermediate levels of amyloid burden who are at the peak of accumulation provides a 4-fold reduction in sample sizes compared to traditional secondary prevention trials (where inclusion criteria includes amyloid-positive individuals regardless of the extent of pathology). As expected, primary prevention trials require larger sample sizes to achieve similar statistical power, but this can be mitigated by targeting inclusion criteria to *APOE*-ε4 carriers and/or by using an early composite region of interest.

First, the direct comparison between dynamic and static parameters in this work confirmed that SUVR largely overestimates DVR and that this bias is strongly dependent on the underlying levels of amyloid burden (Fig. 1a, b). In addition, this overestimation relates to the underlying radiotracer kinetics and can be further influenced by scan time, as well as known confounding effects such as changes in blood flow and tracer clearance [29, 30]. Especially in the case of disease-modifying therapies, an intervention could affect cerebral blood flow and therefore falsely inflate treatment effects when measured by SUVR [31], challenging the interpretation of SUVR-based rates of amyloid accumulation. As a consequence, the results of our primary prevention trial scenario should be interpreted with caution, where the increased accumulation rates observed with SUVR seem to facilitate the detection of treatment effects compared to DVR, despite the increased variability (Tables 1 and 2). Especially in these early stages of disease where the underlying amyloid PET signal is low, the relatively large contribution of physiological and methodologically driven fluctuations in the PET signal can lead to misinterpreted results. This is of particular relevance when the tested intervention may impact cerebral blood flow.

In contrast, secondary prevention trials seem to benefit from the acquisition of dynamic scans, where consistent reductions in sample sizes are observed (Table 2). There, the overestimation of SUVR accumulation rates is less pronounced with respect to its increased variability, resulting in a direct improvement in statistical power when using DVR, a metric with overall lower TRT variability [36]. This finding is in line with a recent publication on tau tracer [^18^F] flortaucipir, where the differences TRT variability between SUVR and *BP*_ND_ also led to smaller sample size requirements when using the latter as quantitative metric [47]. Naturally, obtaining DVR estimates would imply the acquisition of dynamic scans, which can result in a non-negligible increase in patient discomfort, use of scanner time, and overall study cost. To our knowledge, the only available report on the willingness of participants to undergo a second dynamic scan indicates that, at least when using a dual time-window protocol, only 5% of them would consider dropping the study due to discomfort [48]. Further, considering average rates of €750 for a static scan and €1050 for a dynamic scan (available from the AMYPAD Consortium, data not shown), our results indicate that performing dynamic scans may not significantly impact study costs (DVR: *N* = 143, €150 k, SUVR: *N* = 176, €132 k). Therefore, while maintaining similar cost, the acquisition of dynamic scans can increase statistical power, provide additional biomarker information on cerebral blood flow [33, 34], and expose less participants to radiation, an ethical consideration that should not be disregarded [29].

In addition to the increased statistical power of DVR, focusing subject selection in secondary prevention trials to individuals at the peak of amyloid accumulation (20.1 < CL ≤ 49.4) provided a 4-fold reduction in required sample sizes (Table 2). In fact, similar results have been reported by Guo and colleagues, who demonstrated that prevention trials must account for the differences in amyloid accumulation phases (Fig. 2a) by narrowing the range of amyloid burden in inclusion criteria range; otherwise, estimates of treatment effect can be significantly biased [49]. Importantly, the interval of amyloid burden used in our work captures the typical range of amyloid positivity cutoffs derived from visual assessment [45, 50–52], while the upper values around 49.4 CL mostly correspond to levels found in subjects with a clinical presentation of AD [45, 53]. In addition, the range of amyloid burden used in this work for each of the secondary prevention trials are in line to with both the A3 (20–40 CL) and the A45 (CL > 40) trials, both of which target a similar population to the OASIS-3 dataset [11]. Together, our findings further stress the advantages of refining the range of amyloid burden in entry criteria and support the current and future design of smaller, Phase-II, Proof-of-Concept prevention trials in at-risk populations [54]. Of note, these considerations should be weighed against possible higher screening failure rates.

Interestingly, the secondary prevention trial designs tested in this work did not seem to benefit from the use of an early composite ROI. At this stage, the amyloid accumulation in a (global) cortical composite has reached similar rates as those observed in the early regions and has the advantage of larger volume and better count statistics (Table 1). This suggests that, already at the intermediate amyloid burden level, accumulation rates of other regions start to increase and contribute to the global signal. In line with our findings, a previous report described that at higher levels of amyloid burden, the set of regions with increased accumulation rates fall outside of the typical-AD topography [49]. In contrast, primary prevention trials seem to greatly benefit from the use of an early composite ROI, where we observed a ~ 40–50% reduction in expected sample sizes using a ROI composed of precuneus, isthmus cingulate, and lateral orbitofrontal regions (Table 2). These findings are corroborated by a recent report from Insel and colleagues using the Alzheimer’s Disease Neuroimaging Initiative data-set [23]. There, authors showed a reduction of ~ 62% in required sample sizes when using an early ROI composed of precuneus and posterior cingulate. Both early regions proposed by Insel’s and our work, as well as the late ones described by Guo and colleagues are in excellent agreement with recently proposed amyloid burden staging systems [16, 19, 44]. Thus, these findings indicate that in order to significantly impact statistical power, the choice of regions for quantification must be informed by the disease stage of the target population.

Finally, we demonstrated that screening for risk factors such as age and *APOE*-ε4 carriership could further reduce sample size requirements. As expected, age was associated with higher baseline levels of amyloid burden. However, it was not predictive of accumulation rates, which reiterates this is a risk factor for amyloid pathology but does not directly influence the overall accumulation process, as previously suggested in a meta-analysis [13]. Similarly, *APOE*-ε4 carriership was more frequent in subjects with intermediate-to-high amyloid burden, and carriers were younger than their non-carrier counterparts (Table 1). In addition, carriership was only marginally associated with increased accumulation rates, similar to previous work [55, 56], an effect which only reached significance for SUVR (likely due to the proportional bias of this metric which increases for higher levels of amyloid and accumulation rates, see Fig. 1b, d). Together, this suggests *APOE*-ε4 mainly impacts the onset of amyloid pathology rather than the speed of the subsequent accumulation process [57]. These results are in line with several previous reports, which indicate that even in cognitively unimpaired individuals, *APOE* genotype has a substantial effect on the age-related prevalence of AD pathology [13, 58]. In our work, we find that both primary and secondary prevention trials can still significantly reduce required sample sizes when enrolling *APOE*-ε4 carriers alone, despite their younger age. Therefore, enrichment strategies in a general population could focus on older individuals, while specifically targeting *APOE*-ε4 carriers may allow for the inclusion of younger subjects, as these would already have an increased probability of being in the AD continuum. However, such a strategy may impact both screen failure and future labeling of the drug, restricting its prescription from the general population.

It is important to note that all results in this work relate to a fixed effect (20%) of reducing the accumulation rates in amyloid PET scans, which may seem disconnected from the level of amyloid removal observed in recent anti-amyloid immunotherapies [7, 59]. Indeed, most anti-amyloid trials demonstrate such large reductions in amyloid burden that the effects can be appreciated even visually. Nonetheless, other interventions may have more subtle effects on amyloid burden, either directly or indirectly. Some examples would be BACE1 inhibitors [60], drugs with other targets which have downstream amyloid effects [61], or non-pharmacological therapies and multi-domain preventive trials such as those being tested in World-Wide FINGERS [62, 63]. As such, 20% reduction of amyloid accumulation may be a relevant target to detect, especially in a short 1-year Proof-of-Concept study. Nonetheless, the overall sample size impacts of using SUVR/DVR, early/cortical composites, or restricting inclusion criteria can also be observed for larger treatment effects (Supplementary Figure 1). Naturally, these differences become less relevant as the expected reductions become larger.

Methodological issues need to be considered when interpreting the findings of this study. First, while DVR is used as the standard of truth in this work, the chosen imaging window for analysis (30–60 min p.i.) and the use of RLogan could both have affected the results of the comparison between SUVR and DVR. Previous studies have indicated that, prior to the 40–50 min interval, [^11^C]PIB SUV may still be rapidly changing and equilibrium is still not reached. Therefore, this earlier imaging window does not correspond to secular equilibrium conditions, which could have inflated possible flow effects in SUVR and affected RLogan estimates [64]. In addition, RLogan is known to underestimate true binding potential and suffer from noise-induced bias, while other methods such as SRTM2 and MRTM2 have been proposed as optimal for [^11^C]PIB and might have produced higher accumulation rates with DVR [65]. It should also be noted that TRT values from a small single-center study may not translate to the data collected in OASIS-3. However, the differences between SUVR and DVR TRT reported in this work are in line with previous findings with the same tracer [66], as well as with other tracers [47]. Moreover, the TRT dataset analyzed in this work was used as supporting evidence for the superior statistical properties of dynamic PET scans, and the use of literature values would have resulted in equivalent results.

## Limitations

Limitations include the single-tracer character of the study and the relatively limited availability of follow-up data with more than two time points. In addition, one must consider whether the population of OASIS-3 is representative of the primary/secondary prevention trial populations. First, the age range in this work might be too large, but the vast majority of subjects (71%) were between 60 and 85 years of age [11]. Of note, these results may not be comparable to other tracers, as the kinetics of [^11^C]PIB are markedly faster than what is observed with, e.g., the commercially available F-18 tracers such as [^18^F]flutemetamol and [^18^F]florbetaben, which may display even larger biases between SUVR and DVR and therefore also larger differences in sample size requirement between the metrics. This remains to be confirmed and will be explored within the Amyloid Imaging to Prevent Alzheimer’s Disease (AMYPAD) Consortium [67]. Finally, future work in a larger dataset may consider estimating the uncertainty around sample size estimates to better understand the generalizability of these results and relate them to changes in cognitive functioning, which remains the main outcome measure in most preventive trials to date.

## Conclusion

Strategies to improve statistical power differ between secondary and primary AD prevention trials. First, the acquisition of dynamic PET scans can provide reduction in sample sizes only in secondary prevention trials, representing a reasonable alternative to static imaging while reducing the need for exposing healthy participants to ionizing radiation. In contrast, the use of an early composite seem to only benefit primary prevention trials, suggesting that regional analyses must be informed by disease stage in order to provide improved statistical power to trials. Overall, refining inclusion criteria can result in considerable reductions in sample size requirements by identifying individuals at the peak of amyloid accumulation and/or restricting trials to *APOE*-ε4 carriers. These results may provide guidance on how to design smaller Phase II Proof-of-Concept trials without penalizing statistical power to detect treatment-related changes in amyloid accumulation.

## Supplementary Information

**Additional file 1.**

## Data Availability

The datasets analyzed during the current study are available in the OASIS-3 open-access data repository www.oasis-brains.org.
