# Supplementary material for: Strategies to reduce sample sizes in Alzheimer’s disease primary and secondary prevention trials using longitudinal amyloid PET imaging
Source: Alzheimers Res Ther. 2021 Apr 19;13:82. doi: 10.1186/s13195-021-00819-2 (PMC8056524; doi:10.1186/s13195-021-00819-2)
Supplement: Supplementary file 1 — Additional file 1. [file 13195_2021_819_MOESM1_ESM.docx]

**SUPPLEMENTARY METHODS**

Using the LME estimates for annualized accumulation rates and respective standard deviations, the *sampsizepwr* function in Matlab (1-β = 80% power and a two-tailed t-test type-I error of *α*=0.05) was used to determine sample sizes required to detect a range of differences in accumulation rates (0-100% reduction) for a fixed power (80%). The overall trial design assumes participants undergo a PET scan at baseline and another at the completion of the trial. These were computed separately for SUVR and DVR, using the Cortical Composite and the Early Composite, both across the whole population and restricted to *APOE*-ε4 carriers only.

**SUPPLEMENTARY RESULTS**

As per main manuscript, the sample sizes (per arm) were computed for a primary prevention (CL < 20.1), and two secondary prevention trials, one in subjects with intermediate amyloid burden (20.1 < CL ≤ 49.4), and the other in subjects with intermediate-to-high amyloid burden (CL > 20.1).

The overall effects described in the main manuscript are also observed here, where SUVR requires smaller sample sizes for primary prevention, while DVR provides better statistical power for secondary prevention designs. By estimating sample sizes as a function of expected effect, it is also noticeable that these differences are less relevant at larger effects, where sample sizes are similarly small for both metrics (**Supplementary Figure 1**).


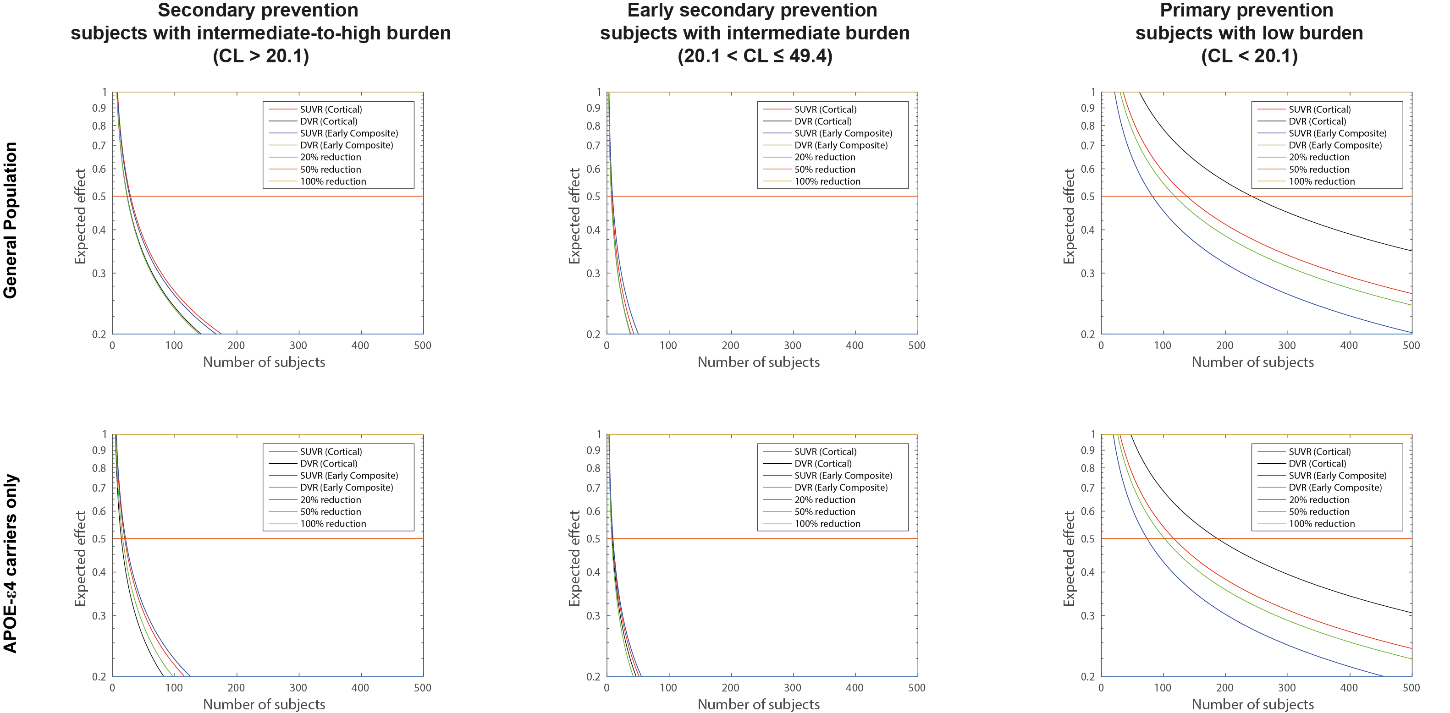


**Figure 1.** *Relationship between sample size (per arm) and expected reductions in accumulation rates (proportional to baseline rates, i.e. y-axis values correspond to proportional reduction, where 0.2 is 20% reduction, 0.5 is 50%, and 1.0 is a complete halt in accumulation).*
